# Ankle Fracture FIXation surgery with or without Tourniquet (AFFIXT): protocol for a randomised controlled feasibility trial

**DOI:** 10.1136/bmjopen-2025-115546

**Published:** 2026-05-12

**Authors:** Muhamed M Farhan-Alanie, Cristiana Huhulea, Meghan Williams, Azra Arif, Prakrit R Kumar, Oliver James Severn Skipper, John McArthur, Jo Parsons, Kate Seers, Xavier Luke Griffin, Julie Bruce

**Affiliations:** 1 Warwick Clinical Trials Unit, University of Warwick, Coventry, UK; 2 University Hospitals Coventry and Warwickshire NHS Trust, Coventry, UK; 3 Barts and The London NHS Trust, London, England, UK; 4 Birmingham Centre for Evidence and Implementation Science (BCEIS), University of Birmingham, Birmingham, UK; 5 Warwick Medical School, University of Warwick, Coventry, UK; 6 Trauma and Orthopaedic Surgery, Queen Mary University of London, Whitechapel, UK

**Keywords:** SURGERY, Foot & ankle, Fractures, Bone, Fractures, Closed, Randomized Controlled Trial, Feasibility Studies

## Abstract

**Background:**

Ankle fracture fixation surgery may be performed with or without the use of a tourniquet. Surgeons who use a tourniquet report reduced intraoperative bleeding, which improves the visualisation of anatomical structures. This may facilitate more accurate fracture fixation and restoration of the anatomical configuration of the ankle joint, potentially leading to improved functional outcomes. An additional proposed benefit of tourniquet use is reduced operative time. In contrast, surgeons who choose not to use a tourniquet report concerns that it may exacerbate postoperative pain and increase the risk of venous thromboembolism, surgical site infection and other complications. However, existing clinical trials are limited by small sample sizes and high risk of bias, preventing the ability to draw robust conclusions. This study aimed to assess the feasibility of conducting a randomised controlled trial (RCT) to evaluate the potential benefits and risks of tourniquet use in ankle fracture fixation surgery.

**Methods:**

This study comprises a two-centre, participant-blinded and surgeon-blinded parallel-arm RCT and an integrated qualitative interview study. A computer-generated randomisation service will allocate up to 50 patients to undergo ankle fracture fixation surgery either with or without the use of a tourniquet. Participants will be followed up for 3 months postoperatively. Primary outcomes include recruitment and retention rates, data completeness, success of blinding and adherence to allocated intervention. Secondary outcomes include postoperative pain, quality of the surgical field, intraoperative blood loss, blood transfusions, procedure duration, skin assessment, awareness of tourniquet use, health-related quality of life (EuroQol-5D-5L), Olerud-Molander Ankle Score and intraoperative and postoperative complications. The integrated qualitative study will consist of semistructured interviews with up to 12 patients and 12 trauma and orthopaedic surgeons (~24 interviews). Interviews will explore perspectives on the feasibility trial, identify factors associated with unblinding and examine barriers and potential solutions to the design and delivery of a future definitive trial. Interviews will be analysed using inductive thematic analysis.

**Ethics and dissemination:**

National Research Ethics Committee (East of England-Essex) approved this study on the 8 May 2025 (REC 25/EE/0051). The results will be disseminated via peer-reviewed publication.

**Trial registration number:**

ISRCTN91783787.

**STRENGTHS AND LIMITATIONS OF THIS STUDY**

- Comprehensive feasibility research.
- Multicentre design.
- Blinding of patients and surgeons (delivering intervention).
- Small sample size.
- Follow-up is limited to 3 months postoperatively.

## Introduction

 Ankle fractures account for approximately 10% of all fractures sustained,^1 2^ with around half of affected patients requiring open reduction internal fixation (ORIF) surgery to restore the anatomical alignment of the ankle joint using various implants. Despite surgical intervention, a substantial proportion of patients report persistent functional limitations, and many are unable to return to their preinjury levels of activity.^3^,^5^ Moreover, complication rates following ORIF for ankle fractures are among the highest in trauma and orthopaedic surgery, with approximately 30% of patients experiencing wound complications, surgical site infection, reoperation, chronic pain or complex regional pain syndrome.^6 7^

Ankle fracture fixation surgery may be performed with or without the use of a tourniquet. This is a cuff device typically placed around the patient’s thigh or calf and then inflated throughout the operation to occlude blood flow to and from the lower leg. According to a 2020 survey of trauma and orthopaedic surgeons, the most commonly cited reasons for tourniquet use are to reduce intraoperative blood loss and improve the visualisation of anatomical structures.^8^ Anecdotally, it is also believed that tourniquet use may facilitate the procedure to be performed more quickly with greater ease and accuracy for fracture fixation and restoration of the anatomical configuration of the ankle joint.^9^ This may optimise patients’ functional recovery and reduce their risk of developing post-traumatic ankle osteoarthritis.^10^ In contrast, some surgeons choose to avoid using a tourniquet, citing concerns that the high pressures exerted on the operative limb may increase patients’ pain both intraoperatively and postoperatively.^11^,^13^ In addition, tourniquet use may increase the risk of venous thromboembolism as a result of arterial and venous stasis. The resulting prolonged ischaemia to the soft tissues, which are already compromised by the initial traumatic injury, can lead to further tissue damage and inflammation, potentially impairing wound healing and increasing the risks of wound-related complications and surgical site infection.^13^,^16^

Although tourniquet use may potentially improve functional outcomes, it may also increase the risk of complications. Previous systematic reviews and meta-analyses of randomised controlled trials (RCTs) have identified up to four tourniquet studies. Across these reviews, the authors reported that definitive conclusions could not be drawn due to small sample sizes and high risk of bias.^12 17 18^ These trials did not evaluate relevant patient-reported (eg, pain intensity, pain character, health-related quality of life (HRQoL) and function) or clinical (eg, analgesia consumption, intraoperative blood loss and quality of the surgical field) outcomes. Review authors recommended further high-quality trials to address this evidence gap. Given the persistent clinical uncertainty surrounding the benefits and risks of tourniquet use in ankle fracture fixation surgery, there is a need for a well-designed multicentre trial addressing the limitations of prior studies and providing robust evidence to inform clinical practice. However, there are several methodological uncertainties that require consideration, including the feasibility of effectively blinding both surgeons and patients to tourniquet use and patient willingness to participate in a clinical trial. Also, many surgeons may be reluctant to alter their established clinical practice for the purposes of research, as views on tourniquet use are ingrained.^8^ Furthermore, the required sample size for a future definitive trial is unknown. Thus, a feasibility trial would evaluate these challenges and determine whether a full definitive trial would be achievable.

**Figure 1:**
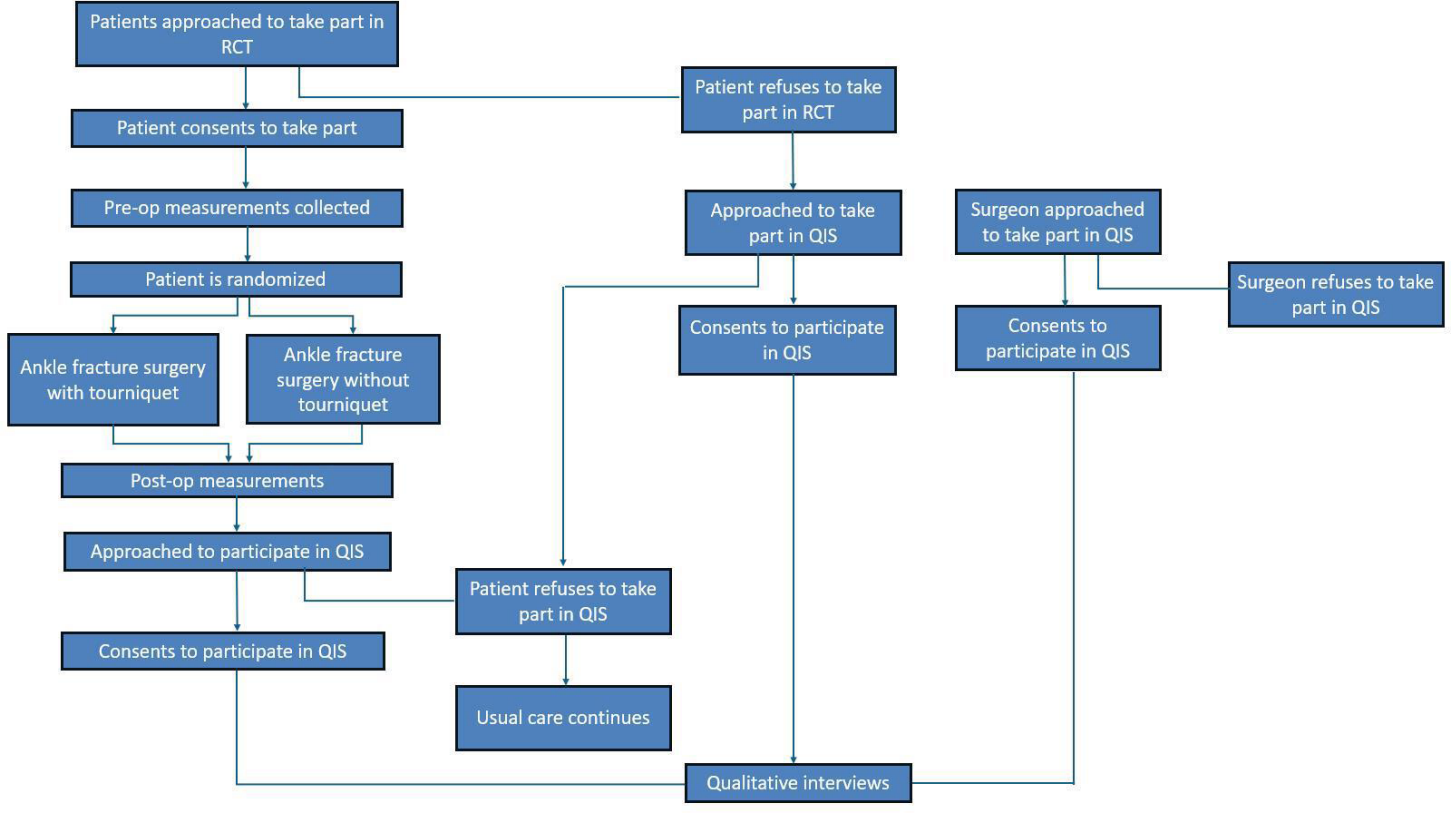
Flow diagram illustrating participant progression through the feasibility randomised controlled trial (RCT) and qualitative interview study. Pre-op, Pre-operatively; Post-op, Post-operatively; QIS, qualitative interview study.

We designed a feasibility study comprising two complementary components: a two-centre feasibility RCT, with an integrated qualitative interview study involving patients and surgeons. The study flow diagram is illustrated in figure 1. The qualitative component is intended to contextualise and enhance the interpretation of the feasibility RCT findings by exploring trial processes in depth, including the perspectives of both patients and surgeons. Together, findings from the RCT and qualitative interviews will inform the design, acceptability and feasibility of the future definitive multicentre RCT.

The aim of this study was to assess the feasibility of conducting a participant-blinded and surgeon-blinded RCT to evaluate the potential benefits and risks of tourniquet use in ankle fracture fixation surgery.

## Methods

### Feasibility RCT

#### Objectives

1. Evaluate the willingness of surgeons to recruit participants.
2. Evaluate the willingness of patients to be randomised.
3. Determine reasons for exclusion of patients.
4. Evaluate the feasibility of blinding surgeons to tourniquet status.
5. Evaluate the feasibility of blinding patients to tourniquet status.
6. Evaluate adherence to trial protocol.
7. Estimate key trial parameters and obtain estimates (of clinical and patient-reported outcome measures) to inform sample size calculation.
8. Determine data completeness, withdrawal rates and optimum time points for follow-up.

#### Reporting

This protocol has been developed in accordance with the Standard Protocol Items: Recommendations for Interventional Trials (SPIRIT) guidelines (online supplemental table 1).^19^ The trial will be reported in line with the Consolidated Standards of Reporting Trials (CONSORT) statement.^20^

Online supplemental table 1 summarises the study design and methods in accordance with the WHO Trial Registration Data Set. The current protocol, V.5.2, was approved on 4 December 2025.

#### Study design and eligibility criteria

This is a multicentre, two-arm, parallel-group feasibility RCT. All patients under the care of trauma and orthopaedic consultants at University Hospitals Coventry and Warwickshire (UHCW) and Barts Health National Health Service (NHS) trusts will be eligible for inclusion, provided they meet the criteria outlined in table 1.

**Table 1:** Trial eligibility criteria

Inclusion criteria	Exclusion criteria
Aged 18 years and over. Patients undergoing primary fracture fixation surgery for a closed ankle fracture within 2 weeks of the date of injury under general and/or regional anaesthetic*. Capacity to give informed consent.	Patients with a pilon fracture rather than an ankle fracture. Unable to understand and speak English. Patients with a chronic musculoskeletal or neurological condition (excluding diabetes mellitus) affecting the operative limb or a history of prior surgery on the affected limb, except for temporary external fixation or manipulation under anaesthetic.

* The requirement for surgery within 2 weeks of injury applied only to participants recruited under protocol versions earlier than V.5.1 (1 December 2025). This restriction was removed following a protocol amendment, which was approved by the sponsor and the Research Ethics Committee before implementation.

#### Participant identification, recruitment and consent

Eligible patients will be approached by a member of the research team either in person or remotely (via telephone or video call) to discuss the study before surgery. Patients will be given a participant information sheet and consent form, along with the opportunity to ask questions. Contact details for the research team will be given to participants. Trial recruitment requires signed written consent.

#### Sample size

No formal sample size calculation is required for a feasibility study. Up to 50 participants will be randomised, similar to other feasibility trials.^21^

Recruitment commenced on 19 June 2025 at UHCW NHS Trust and on 15 August 2025 at Barts Health NHS Trust and concluded on 28 February 2026. Follow-up is expected to be completed in May 2026.

To improve participant recruitment, enrolment will take place across two sites, with systematic identification of eligible patients from admission, clinic and operating lists to minimise missed opportunities. Screening logs will be used to track eligibility and reasons for non-participation, with regular review of recruitment rates and site feedback.

#### Interventions and blinding procedure

Participants will receive anaesthesia in accordance with the standard clinical practice of the attending anaesthetist. Once the participant is fully anaesthetised, one inflatable tourniquet cuff will be applied to either the thigh or calf of the operative limb. This tourniquet will be placed over a thin layer of padding and appropriate isolation measures will be used to prevent skin preparation fluids from seeping beneath the tourniquet cuff, in line with the British Orthopaedic Association Standards for Trauma (BOAST) guidance.^22^

**Figure 2:**
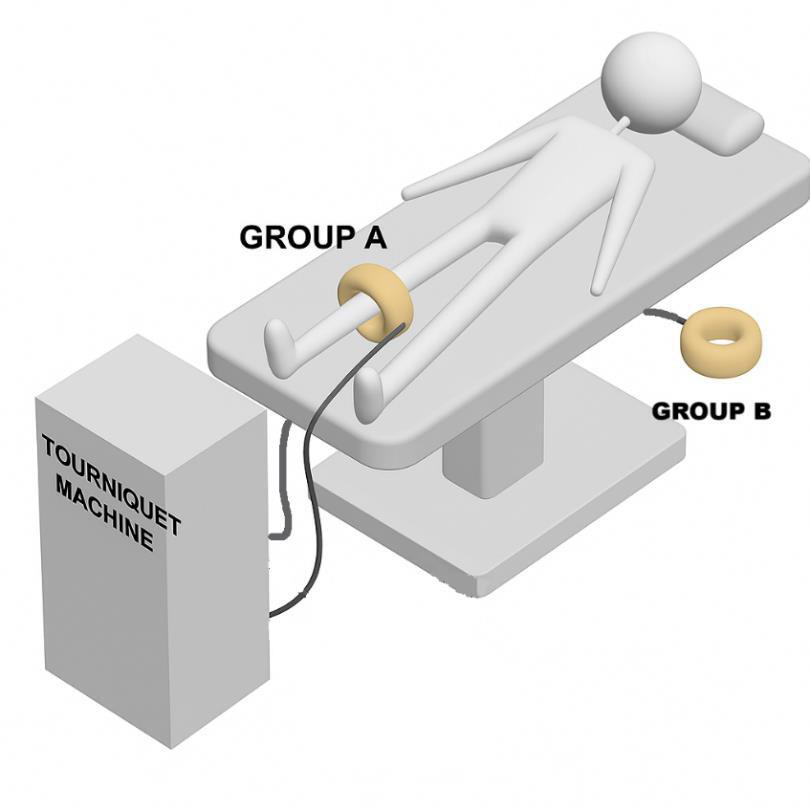
Illustration of participant positioning in theatre. While both tourniquet cuffs are connected to the pneumatic tourniquet machine, only the cuff corresponding to the allocated intervention group will be inflated. Group A, surgery with a tourniquet; Group B, surgery without a tourniquet.

To facilitate blinding, all participants will then have a second inflatable tourniquet cuff applied around a standard hospital pillow, positioned on the floor beneath the operating table and concealed with a standard surgical drape. Application of both these tourniquets will be performed by an appropriately trained individual. Each tourniquet cuff will be connected to a pneumatic tourniquet machine (figure 2), with the machine’s controls and display fully concealed from both the operating surgeons and the participant using standard surgical drapes. Exsanguination of the limb may be achieved by elevating it prior to inflation of the tourniquet.

At the end of the procedure, once the wound has been closed, dressings applied and any backslab or other support applied, the surgeons will be asked to leave the operating theatre and will not be permitted to return until the tourniquet apparatus has been removed by the remaining theatre team. This measure aims to ensure that surgeons are not able to identify any potential marks of tourniquet use on the limb. Surgeons will be informed of the total duration and pressure settings of tourniquet inflation; however, they will not be informed which tourniquet (limb or pillow) was inflated during the operation.

Adherence to delivery of the allocated intervention will be supported through training at the site initiation visit and dedicated intervention training. Concomitant perioperative and postoperative care is permitted as per local standard practice. No prespecified criteria for modification or discontinuation of the interventions were defined. Any intraoperative discontinuation on safety grounds is managed as part of usual clinical care and documented as a deviation.

Unblinding will only be permitted in a clinical emergency where knowledge of the allocated intervention is required to guide emergency clinical care. In that event, the allocation will be revealed by opening the participant’s sealed code-breaking envelope, with completion of an unblinding form and notification of the study team.

#### Randomisation and allocation concealment

Following the application of sterile drapes to the participant, but immediately prior to skin incision, participants will receive one of the following interventions:

- Group A: inflation of the tourniquet applied around the participant’s thigh or calf of the operative limb.
- Group B: inflation of the tourniquet applied around the pillow.

Consented participants will be randomised 1:1 to the intervention (Group A) or the control group (Group B) using a validated online computer-generated sequence via Castor’s Electronic Data Capture (EDC) system, managed by UHCW NHS Trust and will be stratified by study centre.^23^ The randomisation sequence will be created using varying block sizes of 4 and 6 to minimise predictability.

To maintain allocation concealment, randomisation will be performed within 24 hours of the participant’s surgery by an operating department practitioner or another appropriately trained member of the research team. Only the operating department practitioner responsible for implementing the allocation will be informed of the randomisation outcome and is not permitted to disclose it to the participant or any member of the clinical team. The allocation sequence will not be accessible to staff involved in identifying or approaching patients for participation.

#### Inflation procedures

The tourniquet will be inflated to a pressure of 70–130 mm Hg above the participant’s systolic blood pressure, in accordance with BOAST guidance.^22^ Participants will undergo routine ankle fracture fixation using the standard operative technique of the surgeon. The earliest opportunity at which the tourniquet may be deflated is on completion of fracture fixation; however, it may remain inflated beyond this point at the surgeon’s discretion. If the ischaemic tourniquet time exceeds 120 min, the tourniquet must be deflated for a minimum of 10 min. Reinflation is permitted only following a thorough clinical assessment of the relative risks and benefits by the operating surgeon.

#### Clinical outcomes and time points

As this is a feasibility study, the primary outcomes include recruitment and retention rates, data completeness for clinical and patient-reported outcomes, success of blinding and adherence to allocated intervention. Clinical and patient-reported outcomes are not statistically powered and will be summarised descriptively to inform the decision on progression to a future definitive trial.

The secondary outcomes below are also exploratory and not statistically powered. These are intended to inform the selection of outcomes and required sample size for any future definitive trial. They will also provide information on the feasibility of outcome data collection, including questionnaire acceptability and data completeness over follow-up. Participants will receive reminders via phone and/or email to complete outcome questionnaires.

1. Postoperative pain will be assessed at multiple time points, evaluating various attributes, including duration, intensity, location and pain characteristics (eg, neuropathic and nociceptive). Pain location and character will be measured at baseline (preoperatively), 24 hours and 3 months postoperatively using the painDETECT questionnaire.^24^ Pain intensity at the ankle, calf and thigh regions will be assessed using an 11-point Numerical Rating Scale, where 0 represents ‘no pain’ and 10 represents ‘worst possible pain’,^25^ at 24 hours, 3 weeks and 3 months postoperatively. Total inpatient analgesic consumption and medications prescribed or recommended at discharge will be recorded. Data on analgesia use (drug name, dosage, route and timing) will be collected for 3 weeks from surgery.
2. Surgeon-reported quality of the surgical field will be evaluated using a 5-point Likert scale (‘very dissatisfied’, ‘dissatisfied’, ‘unsure’, ‘satisfied’ and ‘very satisfied’).
3. Intraoperative blood loss will be quantified by calculating the difference in weight of the surgical swabs before the skin incision and after wound closure, reported in grams. The volume of fluid collected in the suction container will also be measured. To calculate the volume of blood loss, the volume of any irrigation or other fluids used during the procedure will be subtracted, with the final value reported in millilitres.
4. The number of blood transfusions administered intraoperatively and postoperatively until discharge will be recorded, based on hospital records and reported as the number of units transfused.
5. Intraoperative complications, including vascular, ligamentous, tendinous and neurological injuries, will be captured via a surgeon-completed questionnaire.
6. Postoperative complications will be captured using a self-reported questionnaire, sent to participants at 3 months postoperatively (via email or post). This will include wound complications, surgical site infections, deep vein thrombosis, pulmonary embolism and reoperation (planned and unplanned). Responses will be cross-checked with hospital records, which will also be reviewed to identify additional complications such as fracture non-union, mal-union and neurovascular injury.
7. Tourniquet-related skin injuries, including burns, contusions and pressure sores, will be assessed based on clinical documentation from the operating department practitioners who routinely inspect the tourniquet site before and after its use.
8. Awareness of tourniquet use will be assessed by asking participants and surgeons to complete a short questionnaire on the day of surgery and at 3 months (eg, ‘*Do you think a tourniquet was used during the operation?’*).
9. Procedure duration will be recorded in minutes, from the time of skin incision to completion of wound closure for the ankle fracture fixation procedure.
10. The Olerud-Molander Ankle Score is a validated 9-item patient-reported outcome measure used to assess functional recovery after ankle injuries.^26^ It assesses pain, functional activities and symptoms such as stiffness and swelling, with a total score ranging from 0 to 100 (higher scores indicate better outcomes).
11. HRQoL will be assessed using the EuroQol-5D-5L questionnaire, a self-completed instrument comprising a five-dimensional health status classification system and a visual analogue scale.^27^

Table 2 details the time points at which each outcome will be assessed.

**Table 2:** Time points for assessment of outcomes

Timing	Baseline (retrospective pre injury score)	On day of surgery (pre operatively)	On day of surgery (post operatively)	24 hours post operatively	2 weeks post operatively	3 weeks post operatively	3 months post operatively
Outcome							
Pain (painDETECT)	✓			✓			✓
Pain (NRS)				✓		✓	✓
Analgesia use	✓		✓			✓	
Surgical field of view			✓				
Blood loss			✓				
Blood transfusions			✓		✓		
Length of procedure			✓				
Skin assessment		✓	✓				
Awareness of tourniquet use			✓				✓
EQ-5D-5L	✓					✓	✓
OMAS	✓					✓	✓
Intraoperative complications			✓				
Postoperative complications							✓

EQ-5D-5L, EuroQol-5D-5L; NRS, Numerical Rating Scale; OMAS, Olerud-Molander Ankle Score .

#### Statistical analysis

Categorical variables will be summarised as frequencies and percentages and analysed using χ^2^ or Fisher’s exact tests. Parametric data will be reported using mean and SD and analysed using t-tests. Non-parametric data will be reported as median and IQR and analysed using the Mann-Whitney U test. Effect estimates will be presented with 95% CIs. Given the feasibility design, analyses of clinical outcomes are exploratory and not intended to support definitive inference regarding the effectiveness of the interventions. Statistical tests (p values) will be reported descriptively and interpreted cautiously as the study is not powered to detect between-group differences in clinical outcomes.

### Integrated qualitative interview study

#### Objectives

1. Explore patients’ experiences of participating in the feasibility trial.
2. Explore surgeons’ experiences of participating in the feasibility trial.
3. Explore potential barriers and enablers to patients’ participation and retention.
4. Explore potential barriers and enablers to surgeons’ participation.
5. Explore whether blinding influenced surgeons’ typical practice.
6. Explore the causes of unblinding of patients.
7. Explore the causes of unblinding of surgeons.
8. Understand reasons for any protocol deviation and data incompleteness by surgeons and identify changes to enhance recruitment.
9. Understand reasons for data incompleteness and loss to follow-up of patients and identify changes to enhance recruitment and follow-up.
10. Explore patients’ views on the participant information materials and potential areas for improvement.

#### Reporting

The study will be reported in accordance with the Consolidated Criteria for Reporting Qualitative Research (COREQ).^28^

#### Study design and eligibility criteria

This is a qualitative study involving one-to-one interviews with patients and trauma and orthopaedic surgeons. Patient eligibility criteria match those in the feasibility RCT (table 1). Eligibility criteria for trauma and orthopaedic surgeons are outlined in table 3. Participants will be recruited from the same sites as the feasibility RCT.

**Table 3:** Study eligibility criteria for trauma and orthopaedic surgeons

Inclusion criteria	Exclusion criteria
Aged 18 years and over. Consultant trauma and orthopaedic surgeons who participated or declined to participate in the feasibility RCT. Patients who participated or declined to participate in the feasibility RCT. Ability to provide informed consent.	Unable to understand and speak English

RCT, randomised controlled trial.

#### Participant identification, recruitment and consent

Identification of eligible patients will follow the same process as described in the feasibility RCT methods section. The consent form for the feasibility RCT includes an explicit request for participants’ voluntary permission to be contacted about this qualitative interview study. In the original protocol, patients who provided consent to being contacted could only be approached by telephone or email by a member of the research team after completing their participation in the RCT, to confirm their interest in taking part in the qualitative interview study. Following a protocol amendment (V.5.1, 1 December 2025) patients could be approached 1 month after trial enrolment. Also, patients who declined to participate in the feasibility RCT will have been asked at that time whether they would be willing to participate in this qualitative interview study. In both scenarios, patients will be provided with a participant information sheet and a separate consent form specific to this qualitative interview study. They will also be given the opportunity to ask questions and contact details for the research team will be provided.

Consultant trauma and orthopaedic surgeons based at their participating hospitals will be approached in person or remotely (via phone or email) to explore their interest in being interviewed. The screening log from the feasibility RCT will be reviewed to identify surgeons who took part in or declined involvement in the trial.

Recruitment for both patients and surgeons will require either signed written consent or return of a completed consent form via email, with digital/electronic signatures accepted.

#### Sample size

Approximately 12 trauma and orthopaedic surgeons and 12 patients will be recruited for this study. This sample size is anticipated to be sufficient to achieve data saturation in relation to the study’s objectives.

#### Sampling technique

Purposive sampling will be employed to ensure a diverse range of participant characteristics.

Factors which will be considered during sampling of patients include age, sex, ethnicity and socioeconomic status. Socioeconomic status will be inferred using the Index of Multiple Deprivation, based on the participant’s home postcode.

For trauma and orthopaedic surgeons, sampling will consider ethnicity, sex, years of clinical experience and subspecialty (eg, foot and ankle or trauma).

#### Data collection and analysis

Semistructured interviews will be conducted by surgical researchers, either face-to-face or virtually over Microsoft Teams, depending on participants’ preference. Interviews will be audio-recorded using an encrypted digital voice recorder for in-person interviews or via Microsoft Teams for online interviews. Recordings will be transcribed verbatim, checked for accuracy and anonymised. Transcription will be conducted either by an approved commercial company or through the automated transcription function available in Microsoft Teams. Data will be managed using NVivo qualitative analysis software and analysed using thematic analysis.^29^ Using an inductive approach, data of similar meaning will be grouped into codes, which will then be organised into broader categories by comparing within and across the codes. Overarching themes will be developed to encapsulate these categories. Themes will be explored to ensure they are robust and then explored in relation to relevant theoretical literature. Trustworthiness of the analysis will be achieved through several strategies including peer debriefing and maintenance of a reflexive diary by the lead researcher.^30 31^

### Data management

Each participant will be assigned a unique trial identification number. All RCT data collected will be entered into a secure trial database hosted on Castor’s EDC system.^23^ Identifiable participant information will be stored separately in a locked filing cabinet and linked to the trial data only via the unique trial identification number. Trial records will be audited by the quality assurance team at the Warwick Clinical Trials Unit, in accordance with standard operating procedures.

Audio recording data from the integrated qualitative interview study will be temporarily stored on the University of Warwick OneDrive for Business and a secure link enabling access to these files will be provided to the approved commercial company for transcription. On completion of transcription, data will be deleted from OneDrive for Business.

### Trial oversight

The Trial Management Group (TMG), comprising members of the research team responsible for the day-to-day conduct of the study, will meet monthly. As this is a small feasibility study, there is no independent trial steering committee or data monitoring committee. Adverse events and serious adverse events will be monitored by members of the principal investigator’s trial team. The trial may be stopped prematurely if mandated by the research ethics committee, if a significant and unforeseen safety concern arises or in the event of funding withdrawal. Any proposed amendments to the study protocol will first be reviewed by the TMG and, subject to approval, will be submitted to the trial sponsor, funding body and local research ethics committee for further review and approval. Approved amendments will be communicated to the relevant parties.

### Patient and public involvement

To inform the development of this research and support the grant application, this project was discussed with 10 patients who had undergone ankle fixation surgery. Patients attended an online virtual focus group in December 2020 to discuss their views on tourniquet use in ankle fracture surgery. They said this research project was important to carry out and their views helped shape the design of the research project.

Further engagement work with patients, surgeons and operating department practitioners was completed in preparation for this feasibility trial (REC 23/EE/0214), after the grant was awarded. This included four focus groups involving 20 patients who underwent ankle fracture fixation surgery, completed in 2024. These discussions helped ensure the most important outcomes for patients would be included in this trial. Two patients agreed to act as members for this study’s patient and public involvement group and help contribute to the delivery, management, interpretation and dissemination of this study’s findings.

To inform trial participant eligibility criteria and refine key parameters of tourniquet use, five trauma and orthopaedic surgeons and two anaesthetists were consulted via nominal group technique research. Consensus was obtained on key trial parameters, including patient eligibility criteria, timing of tourniquet inflation and deflation, tourniquet pressures to be used and blinding methodology.

Lastly, four operating department practitioners were interviewed about their role in controlling the tourniquet machine. These interviews helped explore potential blinding methods, identified challenges to maintaining blinding for both patients and surgeons and suggested possible solutions to address these issues. These discussions helped shape this study’s blinding method.

### Trial progress

All necessary approvals are in place and recruitment to both studies is in progress.

### Participant protection and indemnity

No additional ancillary or posttrial care will be provided beyond the usual standard clinical care. Indemnity is in place through the NHS for clinical negligence and through the University of Warwick for harm related to the study design or conduct.

## Supplementary material

10.1136/bmjopen-2025-115546 online supplemental file 1
