# Supplementary material for: Ankle Fracture FIXation surgery with or without Tourniquet (AFFIXT): protocol for a randomised controlled feasibility trial
Source: BMJ Open. 2026 May 12;16(5):e115546. doi: 10.1136/bmjopen-2025-115546 (PMC13182463; doi:10.1136/bmjopen-2025-115546)
Supplement: online supplemental file 1 [file bmjopen-16-5-s001.docx]

Supplementary table 1: Summary of trial information according to the World Health Organization Trial Registration Data Set

| **Data category** | **Information** |
| --- | --- |
| Primary Registry and Trial Identifying Number | ISRCTN registration number 91783787 **(https://www.isrctn.com/ISRCTN91783787)** |
| Date of Registration in Primary Registry | 14^th^ May 2025 |
| Secondary Identifying Numbers | REC reference: 25/EE/0051  IRAS number: 331292  NIHR302653  CPMS 67205  SOC.05/24-25 |
| Source(s) of Monetary or Material Support | National Institute for Health and Care Research |
| Primary Sponsor | University of Warwick, Coventry, CV4 7AL, United Kingdom.  Telephone: 024 7652 3523 |
| Secondary Sponsor(s) | Not applicable |
| Contact for Public Queries | Warwick Clinical Trials Unit, University of Warwick, Coventry, CV4 7AL, United Kingdom.  Telephone: 024 7652 3523  Email: [AffixtStudyOffice@uhcw.nhs.uk](mailto:AffixtStudyOffice@uhcw.nhs.uk) |
| Contact for Scientific Queries | Muhamed Farhan-Alanie  Warwick Clinical Trials Unit, University of Warwick, Coventry, CV4 7AL, United Kingdom.  Telephone: 024 7652 3523  Email: [u1874544@live.warwick.ac.uk](mailto:u1874544@live.warwick.ac.uk) |
| Public Title | Ankle Fracture FIXation with or without Tourniquet (AFFIXT) Study |
| Scientific Title | Tourniquet Use in Ankle Fracture Fixation Surgery: A Feasibility Randomised Controlled Trial |
| Countries of Recruitment | England |
| Health Condition(s) or Problem(s) Studied | Patients undergoing ankle fracture fixation surgery |
| Intervention(s) | Intervention: surgery with the use of a tourniquet  Control: surgery without the use of a tourniquet |
| Key Inclusion and Exclusion Criteria | Refer to Table 2. |
| Study Type | Type: feasibility, interventional, two-centre  Allocation: randomised  Assignment: parallel  Masking: Patients and surgeons |
| Date of First Enrolment | 7^th^ July 2025 |
| Sample Size | Up to 50 |
| Recruitment Status | Trial recruitment closed on 28^th^ February 2026 |
| Primary Outcome(s) | Recruitment rates, retention rates, data completeness, blinding feasibility, and intervention adherence. |
| Key Secondary Outcomes | 1. Post-operative pain (retrospective pre-injury score, and at 24 hours, 3 weeks, and 3 months post-operatively. Analgesia requirements during the hospital admission and at three weeks post-operatively) 2. Surgical field of view (day of surgery post-operatively). 3. Blood loss (day of surgery post-operatively) 4. Blood transfusions (day of surgery and 2 weeks post-operatively) 5. Procedure duration (day of surgery post-operatively) 6. Skin assessment (day of surgery pre- and post-operatively) 7. Awareness of tourniquet use (day of surgery and 3 months post-operatively) 8. Health-related quality of life (EQ5D5L) (retrospective pre-injury score, and 3 weeks and 3 months post-operatively) 9. Olerud-Molander Ankle Score (retrospective pre-injury score, and 3 weeks and 3 months post-operatively) 10. Intra-operative complications (day of surgery post-operatively) 11. Post-operative complications (3 months post-operatively) |
| Ethics Review | National Research Ethics Committee (East of England - Essex) |
| Completion date | 31^st^ May 2026; participant follow-up in progress |
| Summary Results | Participant follow-up in progress |
| IPD sharing statement | Following study completion, deidentified data sets generated will be available on request from WCTU Data Sharing Committee (DSC) ([WCTUDataAccess@warwick.ac.uk](mailto:WCTUDataAccess@warwick.ac.uk)). Access to trial data will require, as a minimum, a data sharing agreement and an ethically approved protocol, alongside any additional conditions stipulated by the Committee. |
