# Supplementary material for: Ankle Fracture FIXation surgery with or without Tourniquet (AFFIXT): protocol for a randomised controlled feasibility trial
Source: BMJ Open. 2026 May 12;16(5):e115546. doi: 10.1136/bmjopen-2025-115546 (PMC13182463; doi:10.1136/bmjopen-2025-115546)
Supplement: Supplementary file 2 [file bmjopen-2025-115546.reviewer_comments.pdf]

PEER REVIEW HISTORY

BMJ Open publishes all reviews undertaken for accepted manuscripts. Reviewers are asked to complete a checklist review form and are provided with free text boxes to elaborate on their assessment. These free text comments are reproduced below.

ARTICLE DETAILS

Title (Provisional)

Protocol for a randomised controlled feasibility trial of tourniquet use in ankle fracture fixation surgery. Ankle Fracture FIXation surgery with or without Tourniquet: the AFFIXT study.

Authors

Farhan-Alanie, Muhamed M; Huhulea, Cristiana; Williams, Meghan; Arif, Azra; Kumar, Prakrit R; Skipper, Oliver James Severn; McArthur, John; Parsons, Jo; Seers, Kate; Griffin, Xavier Luke; Bruce, Julie

---

VERSION 1 - REVIEW

**Reviewer**	**1**
**Name**	**Ponkilainen, Ville**
**Affiliation**	**Central Finland Hospital**
**Date**	**29-Jan-2026**
**COI**	**None**

I would like to thank you for this opportunity to review this trial protocol. The protocol is well written and answers the questions that are needed to evaluate the feasibility of the trial. I appraise the authors for making this effort as a preliminary work before the planned trial.

I have only a few minor remarks for your consideration:

Introduction: Well written, gives the reader a clear message on why this feasibility trial should be done, easy to follow.

Objectives: Unclear under which objective the key secondary outcomes, such as painDETECT, Pain NRS, Olerud-Molander, and HRQoL go? To test whether the patients commit to answer the questionnaires?

Table 1: Some inconsistencies between the protocol and the ICTRP registration:

• Sample size up to 50 vs target sample size is 74

• Date of first enrolment 7th July 2025 vs 19/06/2025

Interventions:

Well described, the way of conducting placebo tourniquet is clever. The only way I think that the surgeon might be unblinded is by evaluating the amount of bleeding perioperatively, but I cannot see how that would be blinded.

Inflation procedures: By deflating the tourniquet during surgery (before the 2 hour time), there is a high chance of unblinding the surgeon; I would consider avoiding that if in the future trial if possible.

Clinical outcomes and time points: "The secondary outcomes below are exploratory and not statistically powered." How about primary outcomes?

Secondary outcomes are not in line with the objectives and are sort of hard to follow. Considering the feasibility point-of-view, I see that the PROM outcomes are used to simulate the real trial, and to evaluate the adherence (missingness of data), instead of comparing the pain, function or HRQoL between two groups. Still the statistical analysis describes the methods to analyse quantitative data and compare two groups and evaluate the treatment effects.

Therefore, the objectives of the feasibility trial are not in line with the statistical analysis section. Please revise the chapter to further describe the qualitative methods that are used or clarify if no such methods are involved.

VERSION 1 - AUTHOR RESPONSE

REVIEWER: 1

Comment 1

I would like to thank you for this opportunity to review this trial protocol. The protocol is well written and answers the questions that are needed to evaluate the feasibility of the trial. I appraise the authors for making this effort as a preliminary work before the planned trial. I have only a few minor remarks for your consideration:

Introduction: Well written, gives the reader a clear message on why this feasibility trial should be done, easy to follow.

Objectives: Unclear under which objective the key secondary outcomes, such as painDETECT, Pain NRS, Olerud-Molander, and HRQoL go? To test whether the patients commit to answer the questionnaires?

Authors' response

The primary objectives of this feasibility study relate to trial processes including recruitment, retention, adherence, and effectiveness of blinding. The patient reported measures (e.g. painDETECT, pain NRS, OMAS, and HRQoL) are included as exploratory secondary outcomes to evaluate the feasibility of outcome collection including acceptability, completion rates, and data completeness at each time point. These measures are also included to generate preliminary estimates to inform the choice of primary outcome and the sample size calculation for a future definitive RCT. These outcomes are not included to confirm treatment effectiveness at this stage. We have clarified this in the manuscript (pages 16 and 21).

Comment 2

Table 1: Some inconsistencies between the protocol and the ICTRP registration:
• Sample size up to 50 vs target sample size is 74
• Date of first enrolment 7th July 2025 vs 19/06/2025

Authors' response

The first point has been addressed under Reviewer 1 above. Regarding the date of first enrolment, this has been corrected on ISRCTN.

Comment 3

Interventions:

Well described, the way of conducting placebo tourniquet is clever. The only way I think that the surgeon might be unblinded is by evaluating the amount of bleeding perioperatively, but I cannot see how that would be blinded.

Inflation procedures: By deflating the tourniquet during surgery (before the 2 hour time), there is a high chance of unblinding the surgeon; I would consider avoiding that if in the future trial if possible.

Authors' response

Thank you for this suggestion. The feasibility trial will evaluate whether blinding of the surgeon is achievable. Immediately post-operatively, surgeons complete a questionnaire asking whether they believe the procedure was performed with or without a tourniquet. Responses

will be used to assess the success of surgeon blinding. Based on these data, we will consider whether it is necessary to limit the surgeon's ability to deflate the tourniquet intra-operatively. However, this may reduce willingness to participate, as some surgeons prefer to confirm haemostasis before tourniquet deflation.

Comment 4

Clinical outcomes and time points: "The secondary outcomes below are exploratory and not statistically powered." How about primary outcomes?

Authors' response

As this is feasibility, neither the primary nor secondary outcomes are statistically powered. Data will be used to determine whether progression to a definitive multicentre RCT is achievable. We have clarified this in the manuscript (page 16).

Comment 5

Secondary outcomes are not in line with the objectives and are sort of hard to follow. Considering the feasibility point-of-view, I see that the PROM outcomes are used to simulate the real trial, and to evaluate the adherence (missingness of data), instead of comparing the pain, function or HRQoL between two groups. Still the statistical analysis describes the methods to analyse quantitative data and compare two groups and evaluate the treatment effects.

Therefore, the objectives of the feasibility trial are not in line with the statistical analysis section. Please revise the chapter to further describe the qualitative methods that are used or clarify if no such methods are involved.

Authors' response

We agree that, in this feasibility study, the patient reported measures are included as exploratory secondary outcomes to determine required sample size, questionnaire completion rates and data completeness across follow up. They are not intended to draw definitive conclusions regarding differences in pain, function, or HRQoL between groups. We have revised the statistical analysis section to clarify that any between group analyses are exploratory (page 21). We have also emphasised that analyses of these outcomes are exploratory and not

intended to support definitive inference regarding intervention effectiveness, and that statistical tests will be reported descriptively and interpreted cautiously, given the feasibility design and limited statistical power. Regarding analysis of the qualitative data, this is already described in the ‘data collection and analysis section’ (page 24).

Thank you again for your helpful review of our manuscript.
